# CSF sTREM2 in neurological diseases: a two-sample Mendelian randomization study

**DOI:** 10.1186/s12974-022-02443-9

**Published:** 2022-04-05

**Authors:** Ming-Hao Dong, Luo-Qi Zhou, Yue Tang, Man Chen, Jun Xiao, Ke Shang, Gang Deng, Chuan Qin, Dai-Shi Tian

**Affiliations:** Department of Neurology, Tongji Hospital, Tongji Medical College, Huazhong University of Science and Technology, Wuhan, 430030 China

**Keywords:** sTREM2, Cerebrospinal fluid, Multiple sclerosis, Mendelian randomization

## Abstract

**Background:**

Soluble triggering receptor expressed on myeloid cells 2 (sTREM2) in cerebrospinal fluid (CSF) has been described as a biomarker for microglial activation, which were observed increased in a variety of neurological disorders.

**Objective:**

Our objective was to explore whether genetically determined CSF sTREM2 levels are causally associated with different neurological diseases by conducting a two-sample Mendelian randomization (MR) study.

**Methods:**

Single nucleotide polymorphisms significantly associated with CSF sTREM2 levels were selected as instrumental variables to estimate the causal effects on clinically common neurological diseases, including stroke, Alzheimer’s diseases, Parkinson’s diseases, amyotrophic lateral sclerosis, multiple sclerosis, and epilepsy and their subtypes. Summary-level statistics of both exposure and outcomes were applied in an MR framework.

**Results:**

Genetically predicted per 1 pg/dL increase of CSF sTREM2 levels was associated with higher risk of multiple sclerosis (OR = 1.038, 95%CI = 1.014–1.064, *p* = 0.002). Null association was found in risk of other included neurological disorders.

**Conclusions:**

These findings provide support for a potential causal relationship between elevated CSF sTREM2 levels and higher risk of multiple sclerosis.

**Supplementary Information:**

The online version contains supplementary material available at 10.1186/s12974-022-02443-9.

## Introduction

In the management of neurological diseases, numerous biomarkers have been identified to improve the accuracy of differential diagnosis and of prognostic assessment. Triggering receptor expressed on myeloid cells 2 (TREM2), which is mainly expressed by microglia, the resident immunocytes in central nervous system (CNS), have been extensively studied and found associated with microglia functions [1]. Evidence from observational studies indicates that the levels of soluble TREM2 (the secreted ectodomain of TREM2, sTREM2) were higher in cerebrospinal fluid (CSF) of patients with CNS disorders of different causes, including inflammatory [2–4], neurodegenerative [5–11], and vascular diseases [12, 13]. However, given careful study design of observational analyses, whether CSF sTREM2 levels account for the observed development of these neurological disorders remains unclear. Disentangling the causal inference of CSF sTREM2 levels and risk of CNS disorders is of great public health and clinical importance.

Mendelian randomization (MR) appears great superiority in investigating causality between traits and diseases, utilizing genetic variants as instrumental variables, which could mimic the coin toss in the randomized trail [14, 15]. Considering the random assignment of alleles in gamete formation, genetic variants could be used to control for confounding as an alternative method. Herein, by leveraging data from the largest scale GWAS on CSF sTREM2 level to date and summary statistics of six common neurological diseases, including stroke, Alzheimer’s diseases (AD), Parkinson’s diseases (PD), amyotrophic lateral sclerosis (ALS), multiple sclerosis (MS), and epilepsy, we implemented a two-sample MR study to investigate the potential causal association between genetic predisposition to CSF sTREM2 levels with risk of diverse neurological diseases and their subtypes.

## Materials and methods

This genetic association study used only published summary data from studies involving human participants, and approved by the Ethics Committee of Tongji Hospital. Strengthening the Reporting of Observational Studies in Epidemiology (STROBE) reporting guidelines were followed. The full period of data collection was June 1, 2021, to July 1, 2021.

### Study design

**Fig. 1:**
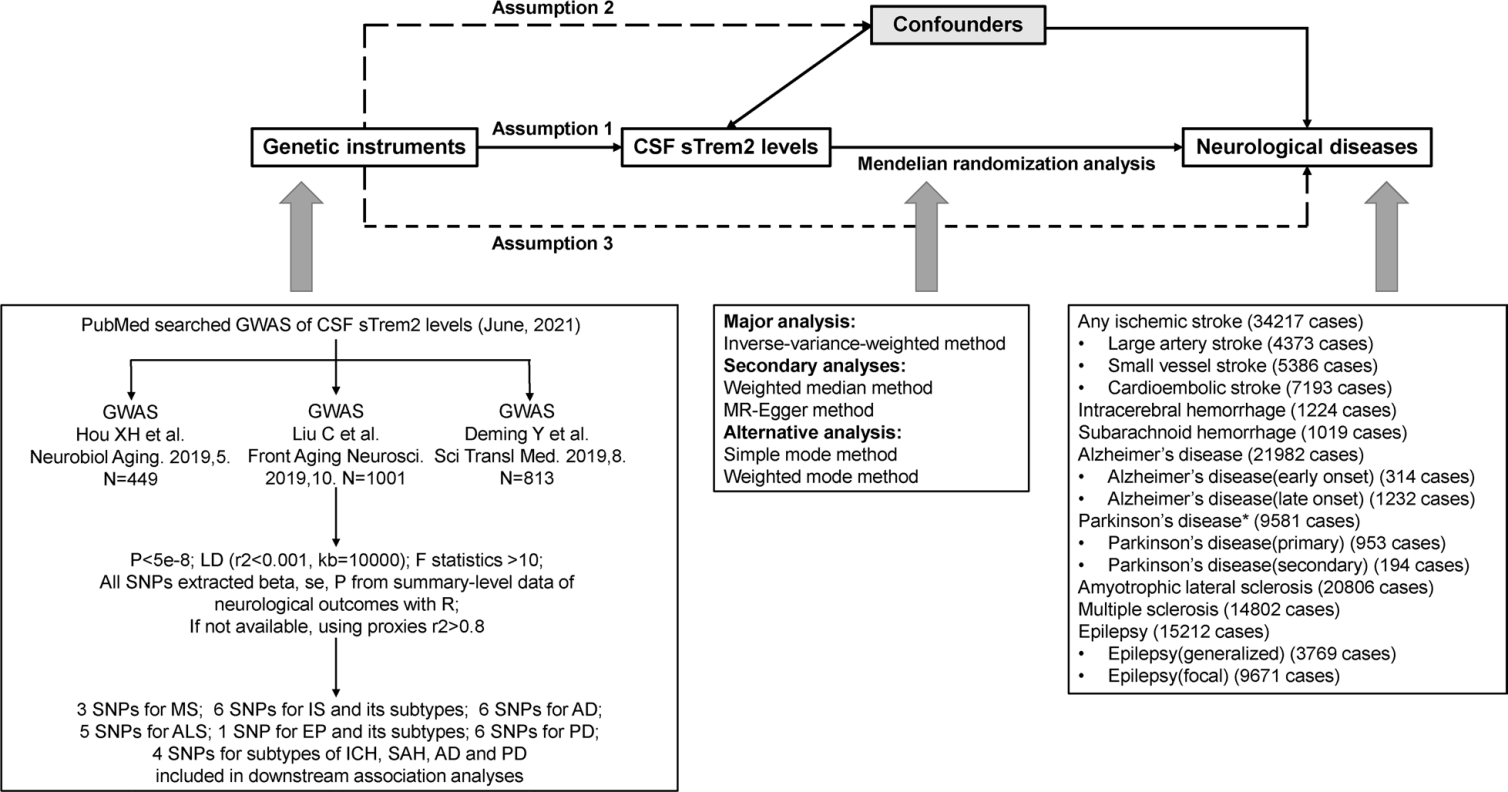
Overview of study design. There exist three significant assumptions for Mendelian randomization (MR). Assumption 1: Genetic instruments should be robustly associated with the exposure. Assumption 2: Genetic instruments should not be associated with any confounders. Assumption 3: Genetic instruments influence risk of the outcome through the exposure, rather than through other pathways. (*CSF* cerebrospinal fluid, *GWAS* genome-wide association study, *LD* linkage disequilibrium, *SNP* single nucleotide polymorphism, *IS* ischemic stroke, *LAA* large artery atherosclerosis, *SVS* small vessel stroke, *CES* cardioembolic stroke, *ICH* intracerebral hemorrhage, *SAH* subarachnoid hemorrhage, *AD* Alzheimer's disease, *PD* Parkinson's disease, *ALS* amyotrophic lateral sclerosis, *MS* multiple sclerosis, *EP* epilepsy)

An overview of the study design and used data sources are displayed in Fig. 1 and Additional file 1: Tables S1, S2. Genetic instruments for CSF sTREM2 levels were selected based on the most recent genome-wide association studies (GWASs) with the largest sample size, including 1001 individuals [16]. Two-sample MR analysis was performed to investigate the association of individual CSF sTREM2-related traits with diverse neurological disorders, including stroke, AD, PD, ALS, MS, and epilepsy. To expel the possibility of reverse causality, we performed a reverse MR analysis to examine the influence of liability to these neurological diseases on CSF sTREM2-related traits.

### Genetic instrument selection

The following procedures were taken to select appropriate genetic variants that preferably satisfy three instrument assumptions of the MR analysis [17]. First, genetic variants, in this case single-nucleotide polymorphism (SNPs), associated with CSF sTREM2 levels were extracted as instrumental variables for corresponding CSF sTREM2-related traits at the genomewide significance level (*p* < 5e−8). Second, a reference panel of 503 Europeans from phase III (version 5) of the 1000 Genomes Project was used for linkage disequilibrium (LD) clumping (*r*^2^ < 0.001) [18]. Third, proxy SNPs having a high correlation coefficient (*r*^2^ ≥ 0.8) based on the European ancestry would take place of the certain SNP which was not available in the summary statistics of the outcome. Fourth, *F*-statistics for each SNP were calculated to quantify the strength of the instruments and weak ones would be excluded (*F*-statistics < 10) [19]. A flow diagram depicting the process of identifying genetic variants to be included in the analyses is shown in Fig. 1.

### Outcome data

The publicly available GWAS summary statistics for different neurological diseases and subtypes were extracted from corresponding authoritative consortium or cohort study. An overview of the used data sources is displayed in Additional file 1: Tables S1, S2.

Summary-level data for ischemic stroke and subtypes were obtained from the MEGASTROKE consortium encompassing 29 genome-wide association studies (GWASs) with a final sample of 440,328 individuals (34,217 ischemic stroke cases and 406,111 non-cases), subtyped according to the Trial of Org 10,172 in Acute Stroke Treatment criteria [20] into large-artery (*n* = 4373), small vessel (*n* = 5386), and cardioembolic (*n* = 7193) stroke [21]. Summary statistics data for intracerebral hemorrhage and subarachnoid hemorrhage were extracted from the FinnGen consortium [22] including 164,757 European-descent individuals (1224 cases and 163,533 controls) and 164,042 European-descent individuals (1019 cases and 163,533 controls) separately (https://finngen.gitbook.io/documentation/).

From the International Genomics of Alzheimer's Project (IGAP), we derived GWAS summary statistics for Alzheimer's disease (21,982 cases and 41,944 controls) [23]. The meta-analysis comprises 4 GWASs, namely, the Alzheimer's Disease Genetics Consortium (ADGC), the Cohort for Heart and Ageing Research in Genomic Epidemiology consortium (CHARGE), the European Alzheimer's Disease Initiative consortium (EADI), and the Genetic and Environmental Risk in Alzheimer's Disease consortium (GERAD/PERADES), in brief. GWAS of AD subtypes were also based on data from the FinnGen consortium, among which 314 individuals with early onset AD and 67,781 healthy controls as well as 1232 individuals with late-onset AD and 67,778 healthy controls were enrolled.

The publicly available GWAS summary statistics for Parkinson's disease included up to 9581 cases and 33,245 controls of European ancestry, which was accessible on the PDGene database [24]. Summary statistics were also acquired from the FinnGen consortium to identify the genetic predisposition of both primary Parkinson's disease (953 cases and 68,589 controls) and secondary parkinsonism (194 cases and 94,467 controls).

Amyotrophic lateral sclerosis summary statistics utilized in this study were collected from a cohort of 20,806 cases and 59,804 controls of European ancestry [25].

Genetic associations with multiple sclerosis were obtained from the data of GWAS on individuals of European ancestry contributed by the International Multiple Sclerosis Genetics Consortium (IMSGC), which included 14,802 cases and 26,703 controls [26].

We further extracted summary estimates of the identified genetic variants from the International League Against Epilepsy (ILAE) Consortium cohort [27]. 44,889 individuals for all epilepsy (15,212 cases and 29,677 controls; ~ 86% Europeans), 33,446 individuals for generalized epilepsy (3769 cases and 29,677 controls) as well as 39,348 individuals for focal epilepsy (9,671 cases and 29,677 controls) were included.

### Statistical analysis

The inverse variance weighted (IVW) method was performed as the major analysis, which provides an estimate with the highest power but is not sensitive to horizontal pleiotropy of instruments. Therefore, the weighted median method and MR-Egger regression were used as secondary methods to correct for pleiotropy. Odd ratios (ORs) and corresponding 95% confidence intervals (95% CI) for outcomes were scaled to 1 pg/dL increase in levels of CSF sTREM2. Cochran’s *Q* statistic and leave-one-SNP-out analysis were conducted to examine the heterogeneity among estimates between each SNP. In addition, simple mode and weighted mode methods were also conducted to assess the robustness of the significant results. All analyses were performed using the TwoSampleMR [28] and MRPRESSO package [29] in R Software 4.0.3 (https://www.R-project.org).

### Data availability

The data sets analyzed in this study are publicly available summary statistics. Data used can be obtained through cited papers and websites.

## Results

**Fig. 2:**
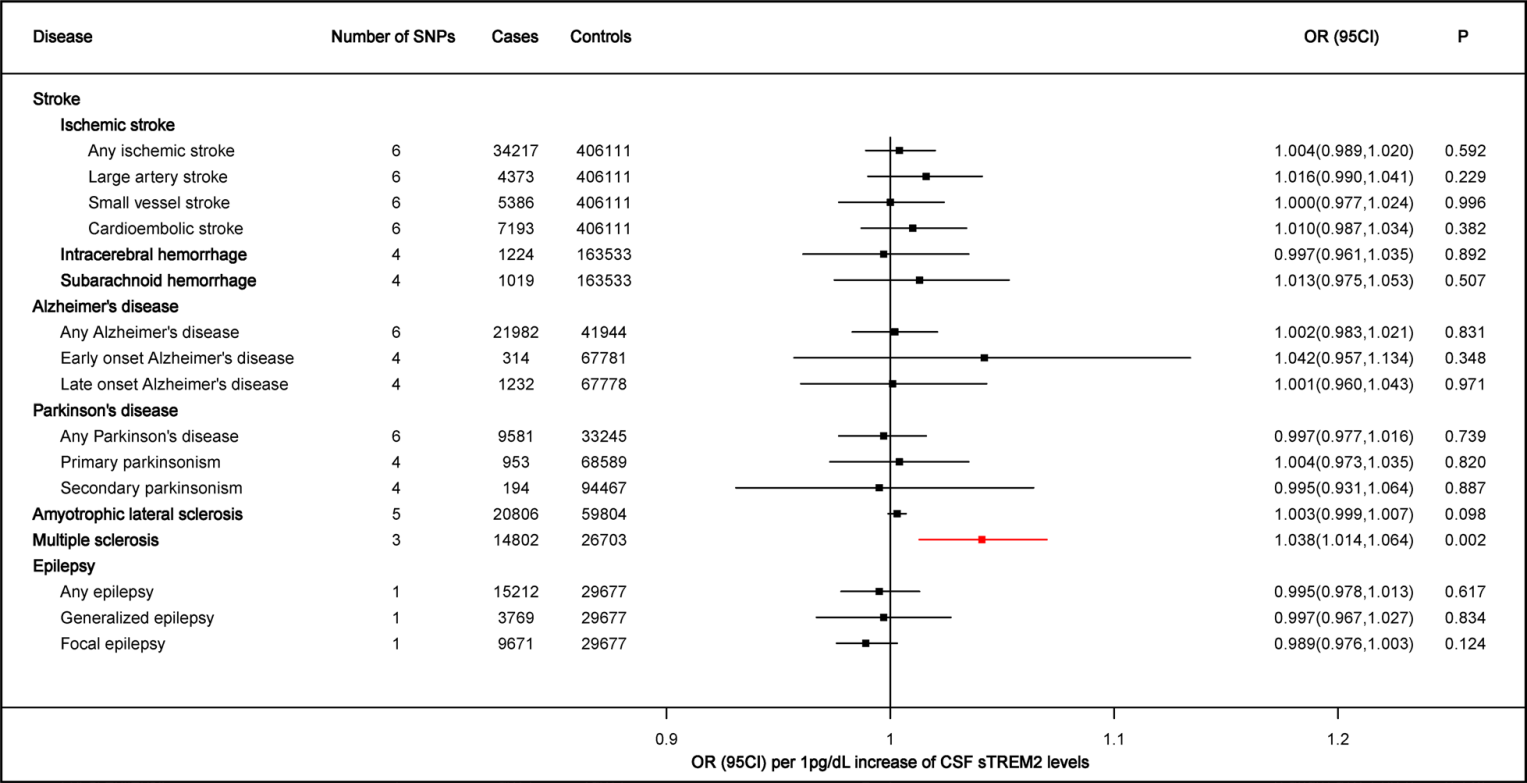
Associations of genetically predicted CSF sTREM2 levels with risk of neurological diseases

**Fig. 3:**
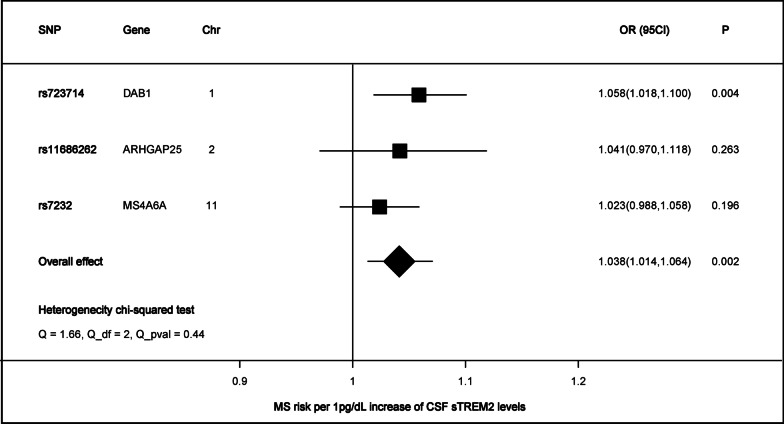
Association between genetically predicted CSF sTREM2 and multiple sclerosis

**Fig. 4:**
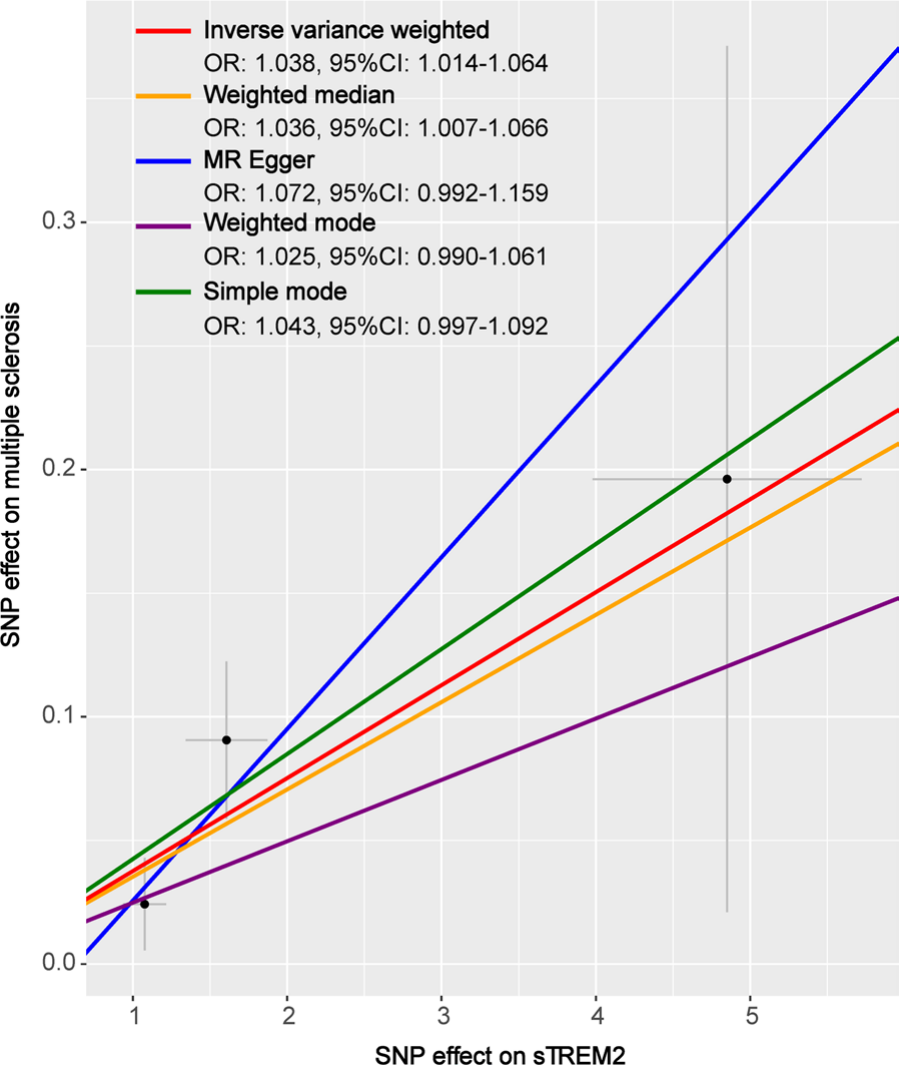
Scatterplot of single nucleotide polymorphism (SNP) potential effects on CSF sTREM2 versus multiple sclerosis, with the slope of each line corresponding to the estimated Mendelian randomization (MR) effect per method

Genetically determined higher CSF sTREM2 levels (1 pg/dL increase) were positively associated with higher odds for multiple sclerosis (OR = 1.038, 95%CI = 1.014–1.064, *p* = 0.002), using the inverse-variance weighted method (Figs. 2, 3). Notably, the effect was detected to be partly driven by rs723714 which located at DAB1(OR = 1.058, 95%CI = 1.018–1.100, *p* = 0.004), as well as rs11686262 mapped at ARHGAP25 and rs7232 mapped at MS4A6A. These three enrolled SNPs could explain 11.2% variance in CSF sTREM2 levels. In addition, results of sensitive analysis indicated no horizontal pleiotropy (*p* values for intercept from MR-Egger > 0.05) or heterogeneity (*p* values for Cochran’s Q statistics > 0.05) existing among the selected instruments (Additional file 1: Table S3). These findings were confirmed using leave-one-SNP-out analysis (Additional file 1: Fig. S1). Exclusion of the outlier did not essentially change the results for CSF sTREM2 with MS. No reverse associations of genetic liability to MS with the levels of CSF sTREM2 (Additional file 1: Table S4). Different MR methods pointed toward the same direction of effect, which suggested a stable causal association in MS. The weighted median estimator, the MR-Egger regression analysis, weighted mode, and simple mode provided estimates of the same magnitude as the fixed-effects IVW analysis for MS (OR = 1.036, 95%CI = 1.007–1.066, *p* = 0.016, OR = 1.072, 95%CI = 0.992–1.159, *p* = 0.331, OR = 1.025, 95%CI = 0.990–1.061, *p* = 0.296 and OR = 1.043, 95%CI = 0.997–1.092, *p* = 0.210, respectively, Fig. 4).

No significant association of genetically determined CSF sTREM2 levels was found with stroke, AD, ALS, PD, epilepsy along with their subtypes (Fig. 2).

## Discussion

The present study for the first time employed MR analysis to evaluate CSF sTREM2 levels as an interested trait with a variety of neurological diseases. Our results provide genetic support for a potential causal association between increased CSF sTREM2 levels and increased risk of multiple sclerosis. There was no evidence for the association between CSF sTREM2 levels and other included neurological disorders along with their subtypes.

Triggering receptor expressed on myeloid cells 2 (TREM2) is a kind of cell surface transmembrane glycoprotein composed of 230 amino acids [30]. Within the CNS, the TREM2 receptors appear to be exclusively expressed by microglial cells [2]. Its expression level varies depending on the cellular context, and the innate immune response and cell signaling pathways can be activated through the combination of its extracellular domain and corresponding ligands [31]. Studies both in vitro and in vivo have shown that the extracellular domain can be cleaved with extracellular release of a soluble TREM2 (sTREM2) fragment which is detectable in CSF and functions independently of TREM2 to regulate interactions between neurons and their surrounding microenvironment [32]. sTREM2 can take up residence around dead neurons, abnormal protein aggregates, or foreign microbes, where it can bind to different receptors to activate innate immune responses and trigger disease conditions [33]. On the other hand, uncontrolled proteolytic cleavage of TREM2 leads to high levels of sTREM2, which can disrupt the blood–brain barrier and result in its diffusion into CSF [34]. This may partly explain the elevated CSF sTREM2 levels in neurodegenerative diseases, such as Alzheimer's disease [35–37], Parkinson's disease [6], as well as amyotrophic lateral sclerosis [11], and neuroinflammatory diseases, such as multiple sclerosis [2, 3]. However, these observational studies can only demonstrate the correlation between CSF sTREM2 levels and the corresponding disease state because of the possibility of bias introduced by confounding factors. Our study, on the other hand, support a potential causal inference of genetically predicted CSF sTREM2 levels for the risk of multiple sclerosis with the aid of the Mendelian randomization framework, in which three selected SNPs strongly associated with CSF sTREM2 levels (*P* < 5e−8) had a similar relation to the risk for multiple sclerosis as the supposedly causal exposure [15].

Multiple sclerosis (MS) is the most common chronic central nervous system (CNS) disorder among young adults, which may lead to severe physical disability in working-age adults. The pathologic characteristics of MS are inflammation, demyelination, glial hyperplasia, and axonal damage in the lesion area [38]. The correlation between sTREM2 and multiple sclerosis was reported back in 2008. The presence of sTREM2 in cerebrospinal fluid was first identified in patients with neuroinflammatory diseases as well as healthy controls [3]. Results showed that sTREM2 in CSF was significantly elevated in individuals with multiple sclerosis and other CNS inflammatory diseases, compared with healthy controls [3]. Another study from Gothenburg confirmed this result and presented that treatment with natazumab or mitoxantrone could normalize the concentration of CSF sTREM2 [2]. This result was again validated in a recent study [4], which further provided evidence of a correlation between CSF sTREM2 levels and T-cell activation, neuroaxonal damage, as well as disease severity. These results suggest that sTREM2 is unlikely to be a diagnostic marker for its lack of specificity, but it may play a role in the pathogenesis of MS [39]. In addition, available evidence indicated that the increased level of sTREM2 in CSF during MS corresponded to the activation of microglia, while the expression level of TREM2 or the activity of the receptor was decreased [2]. Considering that TERM2 receptor may modulate innate immune responses by reducing the primary induction of immune signals to restrain inflammation [2, 40], it seems that activated TREM2 receptor signalling pathway of microglia was possibly inhibited due to ectodomain shedding and lack for ligand binding capacity. Meanwhile, sTREM2 might also act as a soluble "decoy" receptor for endogenous ligands and thus effectively inhibit them binding to the TREM2 receptor. In other words, sTREM2 may be involved in multiple sclerosis by inhibiting the presumed regulatory function of TREM2 ligation on myeloid cells. Our MR result is not only consistent with those of recent reports but also support the potential causal effect of CSF sTREM2 in the risk for MS, due to its great superiority in investigating causality between traits and diseases. Notably, rs723714 as main detected SNP for the effect of MR analysis mapped to Disabled 1 (DAB1), associated with phagocytic activity of microglia [41], indicating its potential role in mechanism of MS via regulation of TREM2 receptor function. In addition, rs7232 mapped to membrane spanning 4-domains A6A (MS4A6A) gene was also detected for the effect of MR analysis. Available studies indicated that MS4A6A and TREM2 were both expressed mainly on microglia [42] and the MS4A gene region was associated with CSF sTREM2 levels [43], which suggested a biological relevance between MS4A6A gene and sTREM2. Nevertheless, the specific role of sTREM2 in the pathogenesis of MS remains to be further clarified. Besides, current MR studies have suggested a causal relationship between inflammatory and immune factors, such as CCL2, NFKB1 [44], IL2Rα [45] and the predisposition to MS, which further indicates that the disorder of inflammatory and immune processes may exert a significant impact on the development of MS, considering the important role of microglia. Yet, the multifactorial pathogenesis of MS requires much more studies to be addressed.

Null causal relationship was estimated between CSF sTREM2 levels and applied diseases except for MS. However, interpretation of negative results must be treated with caution for the reason that they may simply be due to poor statistical power in addition to biological causes.

Numerous studies reported increased CSF sTREM2 levels in Alzheimer's disease, which is a progressive, clinically heterogeneous, and particularly complex neurodegenerative disease characterized by cognitive decline [5, 9, 10, 36, 46, 47]. Pathological features include the accumulation of beta-amyloid protein (Aβ) plaques, intracellular neurofibrillary tangles composed of hyperphosphorylated tau fibers, neuronal degeneration and synaptic loss, neuroinflammation, and glial activation [48]. Accumulating evidences have stated that Aβ, *p*-tau, and *t*-tau in CSF are well performed as disease progression markers [49, 50], while the diagnostic potential of sTREM2 as a biomarker of microglial cell activity in early Alzheimer's disease was also described [47], similar with MS [2]. A significant increase in sTREM2 levels was found at all stages of AD compared to controls, with the highest levels in mild cognitive impairment [46, 47], suggesting that sTREM2 might be a potential biomarker for microglia activity in early stage Alzheimer's disease. Yet, the underlying mechanism remained to be clarified. Notably, CSF sTREM2 was related positively with the above core neurodegenerative biomarkers, such as Aβ42, *p*-tau, and *t*-tau [9, 16], and further results demonstrated that the only factors independently associated with sTREM2 were increasing age and tauopathy [37]. Taking the above information into account, it can be speculated that increased CSF sTREM2 may be due to microglial activation in response to tauopathy. In addition, experiments both in vivo and in vitro suggested that the increased sTREM2 might inhibit Aβ fibrillization and block Aβ-induced neurotoxicity by enhancing microglial survival, proliferation, migration and the uptake and degradation of Aβ [51–53], acting a potential protective role in AD different from in MS. However, this kind of potential advantageous effect was not presented in our MR study, possibly due to the fact that individuals of all stages of AD were enrolled in the exposure statistics, while temporal changes in sTREM2 levels in AD may introduce confounding factors to decrease the validity of genetic instruments [46]. Therefore, an MCI stage-specific GWAS for CSF sTREM2 levels may be better to investigate the function of sTREM2 in the pathogenesis of AD and the embedded molecular mechanism remains to be elucidated.

The most important strengths of our study are its relatively large sample size and the idea of the application of a two-sample MR approach which minimizes the risk of bias from confounding and allowed us to leverage large-scale genetic data on different neurological diseases.

Meanwhile, there are several limitations in this study as well. First, although the largest scale GWAS for CSF sTREM2 levels to date has been included, only 1001 individuals were enrolled due to difficulty of CSF sample collection. In addition, using an AD cohort to estimate the correlation between genetic variants and CSF sTREM2 levels in MS might decrease the validity of instrumental variables considering the potential bias caused from disease type, disease state, age, sex, and etc. Future updated MR analysis using the summary statistics of larger genetic study is warranted to confirm the results of our MR study. Another important limitation of our current study is that we could not directly assess associations of individual genetic variants with potential confounders of association between CSF sTREM2 levels and neurological diseases due to the lack of knowledge of potential confounders and the unavailability of individual-level data. Indeed, potential confounding variables, such as innate immune activation pathways, glial biology, etc., may interfere the results of the feasible causality. Deeper investigation on the functions of selected instrumental variables and more precise algorithm in the MR framework may help eliminate or minimize influences of confounding factors. Third, the CSF sTREM2 level that was measured at a specific timepoint may be affected by many temporary factors, such as age and inflammation, which may not reflect the lifelong CSF sTREM2 level determined by the encoding gene. Fourth, current public database does not contain the summary-level statistics of MS subcategories, making it not possible to further examine the potential relationship between CSF sTREM2 level and risk for subtypes of MS. Fifth, the GWAS data included mainly samples of European, which may limit the generalizability of our findings to other racial/ethnic populations. Finally, although this study suggests that CSF sTREM2 level is causally associated with multiple sclerosis, Mendelian randomization analysis is only a prediction result without verification. Therefore, this kind of causal relationship as well as the underlying pathological mechanism need to be further explored and verified in animal or human experiments.

## Conclusions

A Mendelian randomization study has been conducted to suggest that genetic predisposition to higher CSF sTREM2 levels is associated with higher risk of multiple sclerosis. More attention may need to be paid to explore the potential mechanism of CSF sTrem2 in the pathogenesis of MS, so as to provide a theoretical basis for the proposal of emerging and meaningful therapeutic strategies.

## Supplementary Information

**Additional file 1: Table S1.** Detailed information on used studies. **Table S2.** Detailed information on used studies from FinnGen consortium. **Table S3.** Associations of CSF sTREM2 with neurological diseases and subtypes in inverse-variance weighted method, as well as weighted median method and MR-Egger method. **Table S4.** Effects of genetic liability to MS on CSF sTREM2 levels in reverse Mendelian randomization analysis. **Figure S1.** Leave-one-SNP-out sensitivity analyze for CSF sTREM2 levels on MS.

## Data Availability

All data used in the present study were obtained from genome-wide association study summary statistics which were publicly released by genetic consortia.

## Abbreviations

CSF: Cerebrospinal fluid
sTREM2: Soluble triggering receptor expressed on myeloid cells 2
MR: Mendelian randomization
GWAS: Genome-wide association study
STROBE: Strengthening the Reporting of Observational Studies in Epidemiology
IGAP: International Genomics of Alzheimer's Project
ADGC: Alzheimer's Disease Genetics Consortium
CHARGE: Cohort for Heart and Ageing Research in Genomic Epidemiology consortium
EADI: European Alzheimer's Disease Initiative consortium
GERAD: Genetic and Environmental Risk in Alzheimer's Disease consortium
IMSGC: International Multiple Sclerosis Genetics Consortium
ILAE: International League Against Epilepsy
IVW: Inverse variance weighted
LD: Linkage disequilibrium
SNP: Single nucleotide polymorphism
IS: Ischemic stroke
LAA: Large artery atherosclerosis
SVS: Small vessel stroke
CES: Cardioembolic stroke
ICH: Intracerebral hemorrhage
SAH: Subarachnoid hemorrhage
AD: Alzheimer's disease
PD: Parkinson's disease
ALS: Amyotrophic lateral sclerosis
MS: Multiple sclerosis
EP: Epilepsy
DAB1: Disabled 1
RELN: Reelin
CCL2: Chemokine (C–C motif) ligand 2
NFKB1: Nuclear factor-k-gene binding 1
IL2Rα: Interleukin-2 receptor α subunit
