# Supplementary material for: CSF sTREM2 in neurological diseases: a two-sample Mendelian randomization study
Source: J Neuroinflammation. 2022 Apr 5;19:79. doi: 10.1186/s12974-022-02443-9 (PMC8985278; doi:10.1186/s12974-022-02443-9)
Supplement: Supplementary file 1 — Additional file 1: Table S1. Detailed information on used studies. Table S2. Detailed information on used studies from FinnGen consortium. Table S3. Associations of CSF sTREM2 with neurological diseases and subtypes in inverse-variance weighted method, as well as weighted median method and MR-Egger method. Table S4. Effects of genetic liability to MS on CSF sTREM2 levels in reverse Mendelian randomization analysis. Figure S1. Leave-one-SNP-out sensitivity analyze for CSF sTREM2 levels on MS. [file 12974_2022_2443_MOESM1_ESM.docx]

**Additional information**

**CSF sTrem2 in Neurological Diseases:**

**A Two-sample Mendelian Randomization Study**

Ming-Hao Dong^1#^, Luo-Qi Zhou^1#^, Yue Tang^1^, Man Chen^1^, Jun Xiao^1^, Ke Shang^1^, Gang Deng^1^, Chuan Qin^1*^, Dai-Shi Tian^1*^

**Table S1.** Detailed information on used studies.

**Table S2.** Detailed information on used studies from FinnGen consortium.

**Table S3.** Associations of CSF sTREM2 with neurological diseases and subtypes in inverse-variance weighted method, as well as weighted median method and MR-Egger method.

**Table S4.** Effects of genetic liability to MS on CSF sTREM2 levels in reverse Mendelian randomization analysis.

**Figure S1.** Leave-one-SNP-out sensitivity analyze for CSF sTREM2 levels on MS.

**Table S1**. Detailed information on used studies

| **Exposure/Outcome** | **Data source** | **Participants** | **PubMed ID or web source** |
| --- | --- | --- | --- |
| Summary level data for CSF sTREM2 levels | “Genome-Wide Association Studies for Cerebrospinal Fluid Soluble TREM2 in Alzheimer's Disease” | 1,001 individuals included 224 HN, 72 SMC,  234 EMCI, 277 LMCI, and 194 AD  participants from ADNI. | PubMed ID: 31708768 |
| Genetic instruments for ischemic stroke and subtypes | MEGASTROKE consortium | 440,328 individuals (34,217 ischemic stroke cases and 406,111 non-cases) of European ancestry; 4,373 large artery stroke cases, 5,386 small vessel stroke cases and 7,193 cardioembolic stroke cases | http://www.megastroke.org/ |
| Genetic instruments for ICH/SAH and subtypes of AD/PD | FinnGen consortium | Detailed information is provided  in Supplementary Table 2. | https://finngen.gitbook.io/  documentation/ |
| Genetic instruments for Alzheimer's disease | The NIA Genetics of Alzheimer's Disease Data Storage Site | 21,982 individuals with AD and 41,944 healthy controls of European ancestry | https://www.niagads.org/ |
| Genetic instruments for Parkinson's disease | PDGene database | 9,581 individuals with PD and 33,245  controls of European ancestry | http://www.pdgene.org/ |
| Genetic instruments for amyotrophic lateral sclerosis | “Genome-wide analyses identify KIF5A as a novel ALS gene.” | 20,806 individuals with ALS and 59,804 healthy controls of European ancestry | PubMed ID: 29566793 |
| Genetic instruments for multiple sclerosis | International Multiple Sclerosis Genetics Consortium | 14,802 individuals with MS and 26,703 healthy controls of European ancestry | http://imsgc.net/ |
| Genetic instruments for epilepsy | International League Against Epilepsy Consortium | 44,889 individuals (15,212 epilepsy cases and  29,677 non-cases) of mainly European ancestry (86%); 3,769 generalized epilepsy cases  and 9,671 focal epilepsy cases | https://www.ilae.org/ |

HN, healthy normal; SMC, significant memory concern; EMCI, early mild cognitive impairment, LMCI, late mild cognitive impairment

**Table S2**. Detailed information on used studies from FinnGen consortium

| **Outcome** | **Population** | **Cases** | **Controls** |
| --- | --- | --- | --- |
| Genetic instruments for intracerebral hemorrhage | European | 1,224 | 163,533 |
| Genetic instruments for subarachnoid hemorrhage | European | 1,019 | 163,533 |
| Genetic instruments for Alzheimer's disease (Early onset) | European | 314 | 67,781 |
| Genetic instruments for Alzheimer's disease (Late onset) | European | 1,232 | 67,778 |
| Genetic instruments for primary Parkinson's disease | European | 953 | 68,589 |
| Genetic instruments for Secondary parkinsonism | European | 194 | 94,467 |

**Table S3.** Associations of CSF sTREM2 with neurological diseases and subtypes in inverse-variance weighted method, as well as weighted median method and MR-Egger method.

|  |  |  | **IVW-random effects method** | | | | **Weighted median method** | | | **MR-Egger method** | | | |
| --- | --- | --- | --- | --- | --- | --- | --- | --- | --- | --- | --- | --- | --- |
| **Outcome** | **nSNP** | **Q_pval** | **OR** | **95% CI** | ***p*** | **OR** | | **95% CI** | ***p*** | **OR** | **95% CI** | ***p*** | ***P for intercept*** |
| AIS | 6 | 0.167 | 1.004 | 0.989,1.020 | 0.592 | 1.007 | | 0.992,1.022 | 0.361 | 0.975 | 0.943,1.008 | 0.210 | 0.133 |
| LAA | 6 | 0.394 | 1.016 | 0.990,1.041 | 0.229 | 0.998 | | 0.967,1.030 | 0.895 | 1.035 | 0.965,1.109 | 0.392 | 0.599 |
| SVS | 6 | 0.530 | 1.000 | 0.977,1.024 | 0.996 | 0.991 | | 0.962,1.021 | 0.549 | 0.995 | 0.935,1.058 | 0.870 | 0.858 |
| CES | 6 | 0.232 | 1.010 | 0.987,1.034 | 0.382 | 1.016 | | 0.992,1.041 | 0.187 | 1.029 | 0.965,1.098 | 0.432 | 0.575 |
| ICH | 4 | 0.902 | 0.997 | 0.961,1.035 | 0.892 | 1.006 | | 0.965,1.050 | 0.766 | 0.993 | 0.917,1.076 | 0.882 | 0.916 |
| SAH | 4 | 0.556 | 1.013 | 0.975,1.053 | 0.507 | 1.005 | | 0.955,1.057 | 0.853 | 1.037 | 0.953,1.128 | 0.492 | 0.612 |
| AD | 6 | 0.061 | 1.002 | 0.983,1.021 | 0.831 | 1.000 | | 0.981,1.020 | 0.971 | 1.007 | 0.958,1.059 | 0.795 | 0.838 |
| AD_EO | 4 | 0.051 | 1.042 | 0.957,1.134 | 0.348 | 1.005 | | 0.932,1.083 | 0.900 | 1.009 | 0.810,1.256 | 0.946 | 0.777 |
| AD_LO | 4 | 0.112 | 1.001 | 0.960,1.043 | 0.971 | 1.002 | | 0.961,1.044 | 0.940 | 0.995 | 0.891,1.111 | 0.938 | 0.919 |
| PD | 6 | 0.973 | 0.997 | 0.977,1.016 | 0.739 | 0.998 | | 0.975,1.021 | 0.846 | 1.000 | 0.954,1.049 | 0.987 | 0.872 |
| PD_pri | 4 | 0.876 | 1.004 | 0.973,1.035 | 0.820 | 1.004 | | 0.969,1.040 | 0.840 | 1.015 | 0.950,1.085 | 0.697 | 0.735 |
| PD_scnd | 4 | 0.467 | 0.995 | 0.931,1.064 | 0.887 | 0.985 | | 0.909,1.067 | 0.703 | 0.958 | 0.822,1.117 | 0.642 | 0.642 |
| ALS | 5 | 0.574 | 1.003 | 0.999,1.007 | 0.098 | 1.003 | | 0.999,1.007 | 0.112 | 1.003 | 0.998,1.008 | 0.314 | 0.966 |
| MS | 3 | 0.436 | 1.038 | 1.014,1.064 | 0.002 | 1.036 | | 1.007,1.066 | 0.015 | 1.072 | 0.992,1.159 | 0.331 | 0.554 |
| EP | 1 | \ | 0.995 | 0.978,1.013 | 0.617 | \ | | \ | \ | \ | \ | \ | \ |
| EP_gen | 1 | \ | 0.997 | 0.967,1.027 | 0.834 | \ | | \ | \ | \ | \ | \ | \ |
| EP_foc | 1 | \ | 0.989 | 0.976,1.003 | 0.124 | \ | | \ | \ | \ | \ | \ | \ |

AIS: any ischemic stroke; LAA, large artery atherosclerosis; SVS, small artery stroke; CES, cardioembolic stroke; ICH, intracerebral hemorrhage; SAH, Subarachnoid Hemorrhage; AD, Alzheimer's disease; AD_EO: Early onset Alzheimer's disease; AD_LO: Late onset Alzheimer's disease; PD, Parkinson's disease; PD_pri: Primary parkinsonism; PD_scnd: Secondary parkinsonism; ALS, amyotrophic lateral sclerosis; MS, multiple sclerosis; EP, epilepsy; EP_gen: Generalized epilepsy; EP_foc: Focal epilepsy **Table S4.** Effects of genetic liability to MS on CSF sTREM2 levels in reverse Mendelian randomization analysis.

|  | **Inverse variance weighted method** | | | **Weighted median method** | | | **MR-Egger regression** | | | |
| --- | --- | --- | --- | --- | --- | --- | --- | --- | --- | --- |
| **Outcome** | **Beta** | **SE** | ***p*** | **Beta** | **SE** | ***p*** | **Beta** | **SE** | ***p*** | ***P* for intercept** |
| MS | -0.1621 | 0.1497 | 0.2790 | 0.2611 | 0.0907 | 0.0400 | -0.3795 | 0.8932 | 0.7122 | 0.8269 |

MS, multiple sclerosis; SE, standard error. **Figure S1.** Leave-one-SNP-out sensitivity analyze for CSF sTREM2 levels on MS.


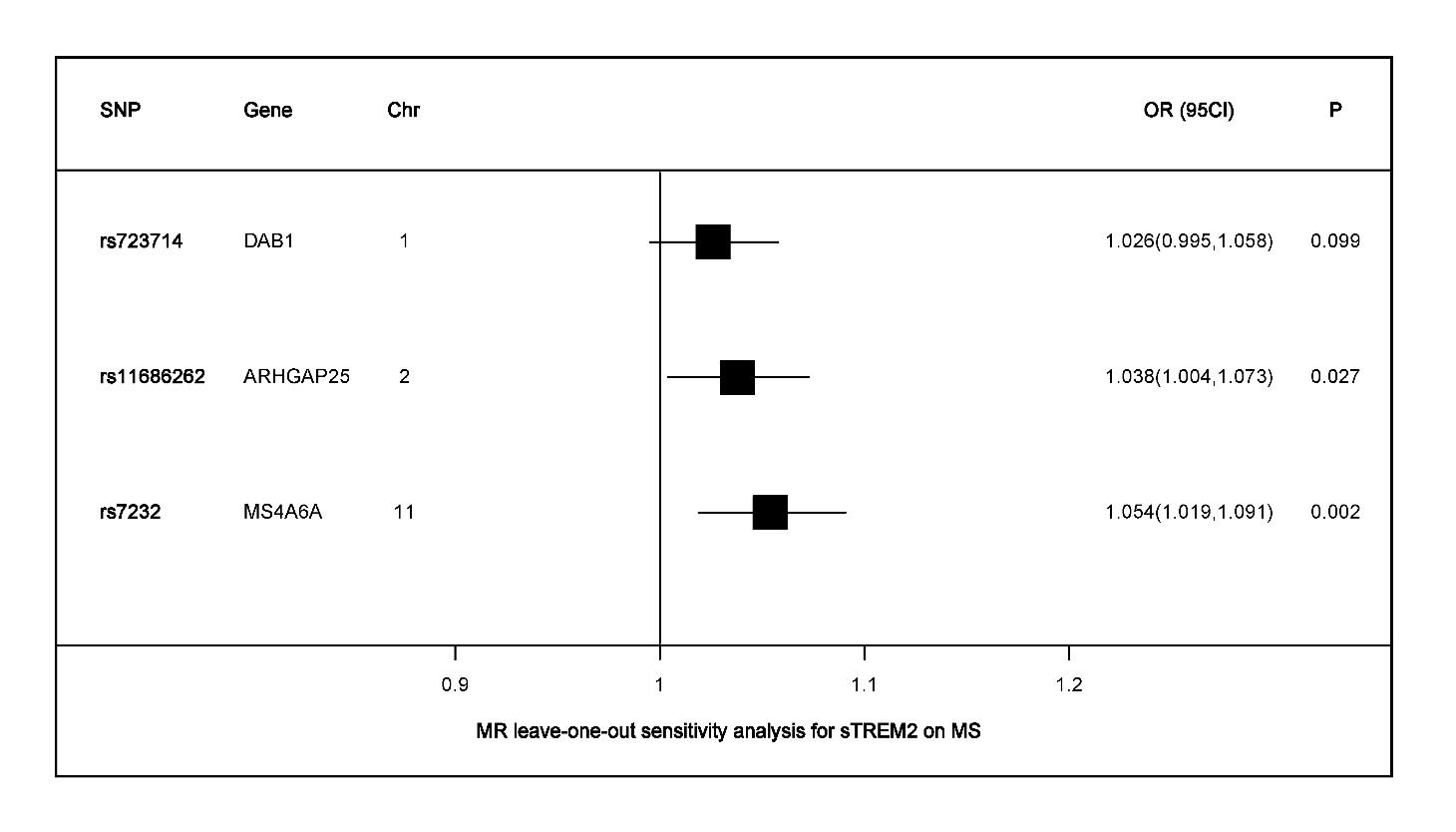


MS, multiple sclerosis
